# Protective role of emodin in rats with post-myocardial infarction heart failure and influence on extracellular signal-regulated kinase pathway

**DOI:** 10.1080/21655979.2021.1983977

**Published:** 2021-11-27

**Authors:** Jinfeng Liu, Liang Ning

**Affiliations:** Department of Cardiovascular Medicine, Avic 363 Hospital, Chengdu, Sichuan Province, China

**Keywords:** Emodin, myocardial infarction, heart failure, energy metabolism, extracellular signal-regulated kinase

## Abstract

We aimed to explore the effects of emodin on the energy metabolism of myocardial cells in rats with post-myocardial infarction (MI) heart failure (HF) and the extracellular signal-regulated kinase (ERK) pathway. The model of MI was established by ligation of the left anterior descending branch. After 4 weeks, the rats with left ventricular ejection fraction (LVEF) of ≤45% were used aspost-MI HF model animals and randomly divided into model, low-dose, middle-dose, high-dose and control groups (n=10). Low-, middle- and high-dose groups were gavaged with 20 mg/kg, 40 mg/kg and 60 mg/kg emodin daily, respectively. After administration for 14 d, the changes in LVEF, left ventricular end-systolic diameter (LVESD), left ventricular end-diastolic diameter (LVEDD) and interventricular septum thickness (IVS) were analyzed. The apoptosis rate of myocardial cells was detected by TUNEL staining. The levels of serum cardiac troponin I (cTnI) and peroxisome proliferator-activated receptor-γ coactivator-1 (PGC-1) were determined using ELISA, and the expressions of mitochondrial respiratory chain complex I protein and phosphorylated-ERK (p-ERK) in myocardial tissues were determined by Western blotting.  Compared with model group, LVEDD, LVESD, apoptosis rate of myocardial cells, levels of serum cTnI and PGC-1, and expressions of complex I and p-ERK in myocardial tissues significantly decreased, while LVEF and IVS increased in low-dose, middle-dose, high-dose and control groups (P<0.05). The changes in the above indices were significantly dependent on the dose of emodin (P<0.05).Emodin can significantly relieve post-MI HF, reduce the apoptosis rate of myocardial tissues, and ameliorate the cardiac function of rats.

## INTRODUCTION

Acute myocardial infarction (MI) with high disability and fatality rates is a common malignant cardiovascular disease. According to a large number of clinical data, post-MI refractory heart failure (HF) is an important cause for death [1]. The clinical symptoms of post-MI HF are decline in cardiac function and dyspnea, with poor prognosis, seriously affecting the quality of life. Moreover, the disease is pathologically characterized by myocardial damage and abnormal apoptosis [2]. Sivasangari *et al*. [3] reported that severe ATP insufficiency in myocardial cells led to ‘energy starvation’ in the myocardium, which was a key event in the abnormal apoptosis of myocardial cells in patients with post-MI HF. Mitochondria are the key organelles supplying energy to cells. Therefore, it is necessary to find suitable drugs for relieving the mitochondrial damage of myocardial tissues and the energy metabolism of myocardial cells in HF patients. Emodin, an anthraquinone derivative and main active component of traditional Chinese medicine rhubarb, has been proven to have antitumor, antibacterial, immunomodulatory and antioxidant activities [4]. It can obviously inhibit the apoptosis of hypoxia/reoxygenation myocardial cells, exerting a protective effect [5]. Extracellular signal-regulated kinase (ERK) signals are activated and phosphorylated after myocardial injury occurs, and involved in regulating myocardial cell growth, apoptosis and other pathological processes [6]. Inhibiting ERK signal transduction can greatly alleviate the mitochondrial damage of myocardial tissues in ischemia-reperfusion rats [7].

In the present study, a rat model of post-MI HF was established, and the effect of emodin on myocardial cells was explored based on ERK signals, aiming to provide new ideas for the clinical treatment of this disease.

## MATERIALS AND METHODS

### Laboratory animals, main reagents and apparatus

A total of 60 SPF male SD rats (10–12 weeks old, 250–280 g) [animal license No. SYXK (Beijing) 2020–0011] were provided by Beijing E-Town Biopharm Generic Technology Co., Ltd., and adaptively fed for 1 week in the SPF animal rooms of our hospital with a light/dark cycle (12 h:12 h) at 22–25°C and 45% humidity. The research purpose and experimental operations had been reported and approved by the Animal Ethics Committee of our hospital.

Emodin (purity >96%) was purchased from Shanghai Aladdin Bio-Chem Technology Co., Ltd. The positive drug Captopril Tablets (25 mg/100 tablets) was manufactured by Guangdong Taicheng Pharmaceutical Co., Ltd. (NMPN H44020939).

Pentobarbital sodium, paraformaldehyde, tissue lysis buffer and enhanced chemiluminescence developing agent (Nanjing Jiancheng Bioengineering Institute), protein concentration assay reagent, Western blotting kits, terminal deoxynucleotidyl transferase-mediated dUTP nick end labeling (TUNEL) staining kits and hematoxylin-eosin (HE) staining kits (Shanghai Beyotime Biotechnology Co., Ltd.), rabbit anti-glyceraldehyde-3-phosphate dehydrogenase (GAPDH) and phosphorylated-ERK (p-ERK) antibodies (Abcam, USA), and JC-1 staining kits (Sigma, USA) were used.

Vevo2100 rat multifunctional ultrasound system (Visual Sonics, Canada), X71 optical microscope (Olympus, Japan), Universal Hood II gel imaging system (Bio-Rad, USA), Philips 201 transmission electron microscope (Eindhoven, the Netherlands), and an ultramicrotome (Leica, Germany) were used.

### Establishment of rat model of post-MI HF

After adaptive feeding, the rat model of MI was established by ligating the left anterior descending coronary artery using the method described by Liu *et al*. [8]: The rats were anesthetized by intraperitoneal injection of pentobarbital sodium (45 mg/kg), and fixed on an operating table in the supine position, with the limbs connected to an electrocardiograph. After skin preparation on the left chest and trachea and disinfection with iodophor, the neck was cut open to expose the trachea, and a ventilator was connected. The chest was cut open horizontally between the left 3rd and 4th intercostal spaces, and the skin was peeled off bluntly until the middle area between the left atrial appendage and pulmonary artery, i.e. the left anterior descending coronary artery, was exposed. Then, it was ligated with #6-0 surgical suture. When the myocardium below the ligation site gradually turned from red to gray white, thoracic congestion was removed, and the chest was sutured with #3 suture layer by layer and closed. After autonomous respiration was restored, ST segment elevation shown in the electrocardiogram indicated successful establishment of the MI model. The rats were intramuscularly injected with penicillin for 5 consecutive days after operation to prevent infection. Four weeks after operation, cardiac function ultrasonography was performed, and the rats with left ventricular ejection fraction (LVEF) of ≤45% were used as post-MI HF model animals.

### Animal grouping

The post-MI HF model was successfully established in 50 rats which were divided into model, low-dose, middle-dose, high-dose and control groups (n = 10) using a random number table. Another 10 rats were used as sham group, and they were only threaded but not ligated during modeling. After successful modeling, low-, middle- and high-dose groups were gavaged with 20 mg/kg, 40 mg/kg and 60 mg/kg emodin daily, respectively, control group was gavaged with 10 mg/kg captopril, while sham and model groups were gavaged with an equal dose of normal saline. All rats were fed with basic feed in separate cages, and drank water freely.

### Changes in cardiac function

After drug administration for 14 d, the rats received induction of anesthesia and heart color Doppler ultrasonography, and LVEF, left ventricular end-systolic diameter (LVESD), left ventricular end-diastolic diameter (LVEDD) and interventricular septum thickness (IVS) were analyzed. The ratio of intensity of red fluorescence to that of green fluorescence was used to indicate the mitochondrial membrane potential of myocardial tissues [9].

### Apoptosis of myocardial tissues

The myocardial tissues of rats were pretreated according to the instructions of TUNEL staining kits, subjected to antigen retrieval, blocked, labeled, stained in dark, dehydrated, and mounted with neutral balsam, followed by microscopic examination. The cells stained yellowish brown in the field of view were apoptotic, and the ratio of number of TUNEL-positive cells to that of total cells indicated the apoptosis rate [10].

### Changes in serum myocardial energy metabolism factors cardiac troponin I (cTnI) and peroxisome proliferator-activated receptor-γ coactivator-1 (PGC-1)

The serum of rats was centrifuged, and the levels of serum cTnI and PGC-1 were detected by enzyme-linked immunosorbent assay (ELISA) according to the instructions of kits [11].

### Expressions of mitochondrial respiratory chain complex I protein and p-ERK in myocardial tissues

The myocardial tissues of rats were placed in lysis buffer at 4°C for 30 min and centrifuged, and the supernatant was diluted, from which total protein was routinely extracted. About 50 μg of samples were loaded, subjected to electrophoresis, transferred onto a membrane and blocked. After incubation with the primary antibodies (1:1000) and secondary antibodies (1:5000) at room temperature, the color was developed for 30 min. With GAPDH as a reference, the gray value of target protein was analyzed [12].

### Statistical analysis

SPSS 16.0 software was used for statistical analysis, and GraphPad Prism 8.0 was used for plotting. Normally, distributed data such as the cardiac function parameters were expressed as mean ± standard deviation ($\bar{X}$_±*s*_). Univariate analysis was performed for comparisons among groups, and *t* test was conducted for pairwise comparison. P < 0.05 was considered statistically significant.

## RESULTS

### General state of rats

During the experiment, the rats in sham group had a good mental state, many activities, quick response, and soft and shiny fur. The ears and paws of rats were ruddy and had no edema, there were no abnormalities in food or water intake, and the increase in body weight was obvious. In model group, the rats were in an obvious low spirit, frequently curled up, and almost had no activity or slow response. The fur was dark and messy with obvious hair loss, nasolabial cyanosis occurred, and some rats had obvious edema in both ears, four paws or tail, poor appetite, and insignificant increase in body weight. In low-, middle- and high-dose groups and control group, the symptoms in mental state, reactivity, fur, water intake, paws and ears were alleviated to different extents. In particular, the increase in body weight tended to be the same in high-dose group, control group and sham group. Collectively, the drug had apparent intervention effects on the symptoms of rats.

### Changes in cardiac function

Discovering drugs from the rich resources of traditional Chinese medicine that can effectively protect against myocardial ischemia-reperfusion injury is of great clinical value. As the main active ingredient of traditional Chinese medicine rhubarb, emodin can scavenge free radicals, as well as exert antioxidative, anti-inflammatory, antitumor and anti-apoptotic effects [13]. At present, the effects of emodin on post-MI HF remain largely unknown, and the mechanism is still unclear.

**Figure 1.**
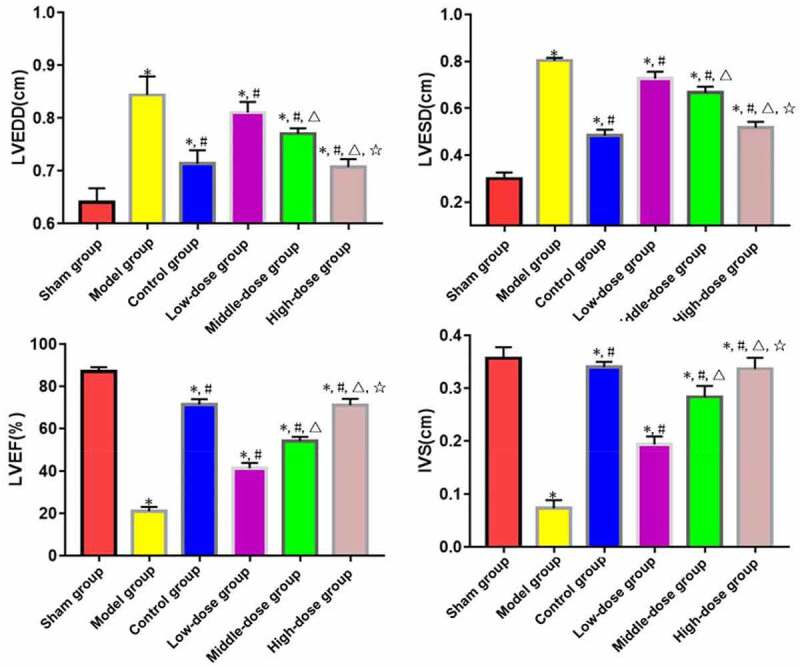
Cardiac function indices. *P < 0.05 *vs*. sham group, ^#^P < 0.05 *vs*. model group, ^∆^P < 0.05 *vs*. low-dose group, ^☆^P < 0.05 *vs*. middle-dose group

LVEDD and LVESD significantly increased, while LVEF and IVS decreased in model, low-dose, middle-dose, high-dose and control groups compared with those in sham group (P < 0.05). The opposite results were found in low-dose, middle-dose, high-dose and control groups compared with those in model group (P < 0.05). Moreover, the changes in LVEDD, LVESD, LVEF and IVS were obviously dependent on the dose of emodin (P < 0.05), without significant differences between high-dose and control groups (P > 0.05) (Figure 1).

### Apoptosis of myocardial cells

HF patients have obvious cardiomyocyte apoptosis, accompanied by scarce microvessels, smooth muscle remodeling of small blood vessels and vascular cell apoptosis. How to terminate or reverse the process of cell apoptosis upon HF has become the key to the improvement of prognosis [14].

**Figure 2.**
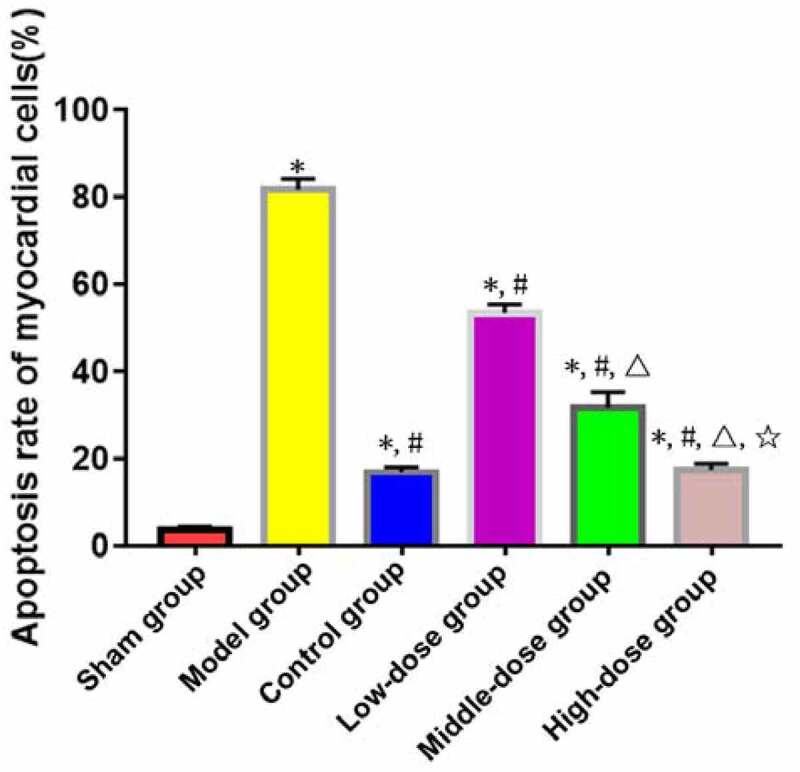
Apoptosis of myocardial cells (×400). *P < 0.05 *vs*. sham group, ^#^P < 0.05 *vs*. model group, ^∆^P < 0.05 *vs*. low-dose group, ^☆^P < 0.05 *vs*. middle-dose group

TUNEL staining exhibited that the apoptosis rate of myocardial cells was significantly higher in model, low-dose, middle-dose, high-dose and control groups than that in sham group (P < 0.05). The apoptosis rate of myocardial cells was significantly lower in low-dose, middle-dose, high-dose and control groups than that in model group (P < 0.05). Moreover, the changes in the apoptosis rate of myocardial cells were obviously dependent on the dose of emodin (P < 0.05), without significant difference between high-dose and control groups (P > 0.05) (Figure 2).

### Serum contents of cTnI and PGC-1

CTnI is an ideal cardiomyocyte-specific marker, and also one of the crucial indicators for the clinical diagnosis of myocardial injury. It is a regulatory protein that only exists in the myocardium and plays an essential role in myocardial contraction and relaxation [15]. Recently, mitochondrial production has been closely related to the regulation of mitochondrial functions, of which PGC-1 may be a key regulator. Especially, in the cardiovascular system, mitochondrial production regulated by the PGC-1 signaling pathway may be one of the main mechanisms for maintaining and repairing the mitochondrial function of cardiomyocytes and vascular endothelial cells, dominating in the onset and progression of cardiovascular diseases such as HF, myocardial hypertrophy and diabetes-induced cardiovascular complications [16].

**Figure 3.**
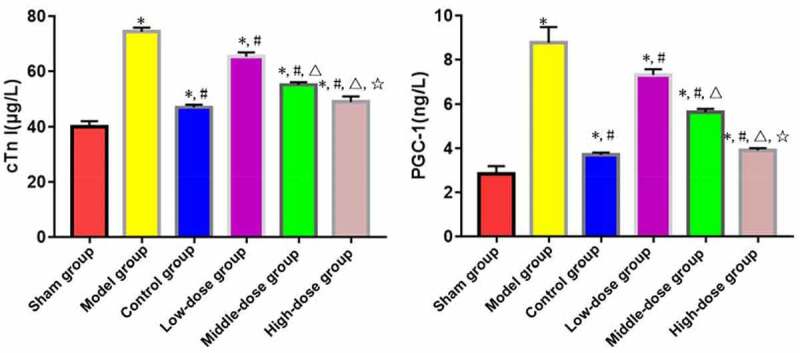
Serum contents of cTnI and PGC-1. *P < 0.05 *vs*. sham group, ^#^P < 0.05 *vs*. model group, ^∆^P < 0.05 *vs*. low-dose group, ^☆^P < 0.05 *vs*. middle-dose group

ELISA revealed that model, low-dose, middle-dose, high-dose and control groups had significantly increased levels of serum cTnI and PGC-1 compared with those of sham group (P < 0.05). Low-dose, middle-dose, high-dose and control groups had significantly decreased levels of serum cTnI and PGC-1 compared with those of model group (P < 0.05). Moreover, the changes in content of serum cTnI and PGC-1 were obviously dependent on the dose of emodin (P < 0.05), without significant differences between high-dose and control groups (P > 0.05) (Figure 3).

### Expressions of complex I and p-ERK in myocardial tissues

**Figure 4.**
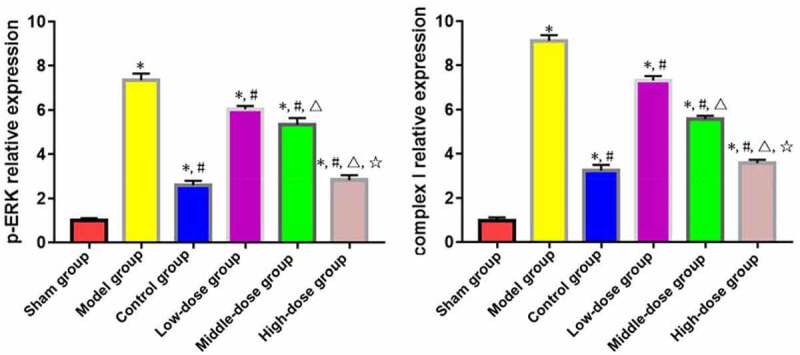
Expressions of complex I and p-ERK in myocardial tissues. *P < 0.05 *vs*. sham group, ^#^P < 0.05 *vs*. model group, ^∆^P < 0.05 *vs*. low-dose group, ^☆^P < 0.05 *vs*. middle-dose group

According to the results of Western blotting, the expressions of complex I and p-ERK in myocardial tissues significantly rose in model, low-dose, middle-dose, high-dose and control groups compared with those in sham group (P < 0.05). The opposite results were found in low-dose, middle-dose, high-dose and control groups compared with those in model group (P < 0.05). Moreover, the changes in expressions of complex I and p-ERK in myocardial tissues were obviously dependent on the dose of emodin (P < 0.05), without significant differences between high-dose and control groups (P > 0.05) (Figure 4).

## DISCUSSION

Chronic HF is the end stage of cardiovascular diseases such as myocarditis, hypertension, hyperlipidemia, acute MI and diabetes. Among them, acute MI accounts for about 60–70% of HF, which seriously affects the normal life of patients, brings heavy economic and mental burdens to their families, and poses severe challenges to the sustainable development of social public health undertakings. It has been confirmed in a large number of studies that insufficient myocardial energy supply caused by MI is an important cause for abnormal myocardial apoptosis and changes in myocardial structure inducing cardiac dysfunction [17]. Therefore, it is necessary to clarify the molecular mechanism underlying the energy metabolism of myocardial cells, and to seek effective intervention methods for alleviating the myocardial energy imbalance after myocardial damage and inhibiting the changes in myocardial structure, which can help improve the cardiac function and quality of life of patients after MI.

Traditional Chinese medicine rhubarb acts on the spleen, stomach, liver and heart meridians, and has medicinal values of detoxicating, promoting blood circulation, clearing heat, eliminating congestion by purgation, reducing internal heat and resolving phlegm. As a natural anthraquinone derivative, emodin is the most important active component of rhubarb, which can well improve the microcirculation, resist bacteria, tumors and inflammation, and exert liver-kidney and cardiovascular protective effects. Ye *et al*. [5] showed that emodin significantly inhibited apoptosis caused by hypoxia/reoxygenation, reduced the proportion of MI in rat models of ischemia-reperfusion injury, and ameliorated the cardiac function of animals. Zhang *et al*. [18] found that emodin obviously relieved mitochondrial ultrastructural damage caused by inflammatory stress, abnormal apoptosis of endothelial cells and vascular endothelial dysfunction, verifying its high value for the drug development for early cardiovascular diseases such as hypertension. Su *et al*. [19] confirmed that emodin markedly regulated the expressions of lipid metabolism factors such as proprotein convertase subtilisin/kexin type 9 to alleviate high-fat diet-induced lipid accumulation in the myocardial tissues of rats, indicating that emodin can well regulate lipid metabolism. However, the effect of emodin on post-MI HF remains unclear yet. In the present study, compared with model group, the clinical symptoms of rats were significantly alleviated, the pathological and ultrastructural damage of myocardial tissues was significantly relieved, the apoptosis rate declined, and the mitochondrial membrane potential was restored in low-, middle- and high-dose groups, confirming the therapeutic effect of emodin.

Mitochondria are organelles for energy metabolism, and the respiratory chain enzyme complexes in the inner mitochondrial membrane are key components involved in mitochondrial energy metabolism. ERK protein widely participates in regulating the growth, proliferation and apoptosis of myocardial cells. After myocardial damage occurs, extracellular signals are transferred by ERK protein into cells, and phosphorylated before entering the cell nucleus, thus enhancing the transcription and translation of inflammatory factors and oxidative stress molecules, and leading to mitochondrial structural changes, decline in mitochondrial membrane potential, energy metabolism disorders and abnormal myocardial apoptosis [20]. As a result, cardiac structure is altered, and cardiac dysfunction is obvious. Zhang *et al*. [21] showed that in the rat model of septic cardiomyopathy, suppressing the phosphorylation of ERK markedly alleviated lipopolysaccharide-induced mitochondrial damage, improved the mitochondrial function, and relieved the cardiac dysfunction in animals during modeling. In this study, the levels of cTnI and PGC-1 (specific markers directly reflecting the energy metabolism of myocardial cells), and the expressions of complex I and p-ERK in myocardial tissues declined with rising dose of emodin, confirming the target of this drug.

## CONCLUSION

In conclusion, emodin can significantly improve energy metabolism, reduce the apoptosis rate of myocardial tissues, and ameliorate the cardiac function of post-MI HF rats. However, whether emodin also affects the progression of disease through other targets remains to be explored by more in-depth studies.

## Highlights

- Emodin significantly relieves post-myocardial infarction heart failure.
- Emodin reduces the apoptosis rate of myocardial tissues.
- Emodin ameliorates the cardiac function.

## References

[1] Jenča D, Melenovský V, Stehlik J, et al. Heart failure after myocardial infarction: incidence and predictors. ESC Heart Fail. 2021;8(1):222–237. 33319509 10.1002/ehf2.13144 PMC7835562

[2] Molitor M, Rudi WS, Garlapati V, et al. Nox2+ myeloid cells drive vascular inflammation and endothelial dysfunction in heart failure after myocardial infarction via angiotensin II receptor type 1. Cardiovasc Res. 2021;117(1):162–177. 32077922 10.1093/cvr/cvaa042

[3] Sivasangari S, Asaikumar L, Vennila L. Arbutin prevents alterations in mitochondrial and lysosomal enzymes in isoproterenol-induced myocardial infarction: an in vivo study. Hum Exp Toxicol. 2021;40(1):100–112. 32757845 10.1177/0960327120945790

[4] Dong X, Fu J, Yin X, et al. Emodin: a review of its pharmacology, toxicity and pharmacokinetics. Phytother Res. 2016;30(8):1207–1218. 27188216 10.1002/ptr.5631 PMC7168079

[5] Ye B, Chen X, Dai S, et al. Emodin alleviates myocardial ischemia/reperfusion injury by inhibiting gasdermin D-mediated pyroptosis in cardiomyocytes. Drug Des Devel Ther. 2019;13:975–990. 10.2147/DDDT.S195412 PMC6438141 30988600

[6] Yang Y, Ding Z, Zhong R, et al. Cardioprotective effects of a Fructus Aurantii polysaccharide in isoproterenol-induced myocardial ischemic rats. Int J Biol Macromol. 2020;155:995–1002. 31712158 10.1016/j.ijbiomac.2019.11.063

[7] Fu Z, Jiao Y, Wang J, et al. Cardioprotective role of melatonin in acute myocardial infarction. Front Physiol. 2020;11:366. 32411013 10.3389/fphys.2020.00366 PMC7201093

[8] Liu Z, Tao B, Fan S, et al. Over-expression of microRNA-145 drives alterations in β-adrenergic signaling and attenuates cardiac remodeling in heart failure post myocardial infarction. Aging (Albany NY). 2020;12(12):11603–11622. 32554856 10.18632/aging.103320 PMC7343449

[9] Song Y, Wang B, Zhu X, et al. Human umbilical cord blood–derived MSCs exosome attenuate myocardial injury by inhibiting ferroptosis in acute myocardial infarction mice. Cell Biol Toxicol. 2021;37(1):51–64. 32535745 10.1007/s10565-020-09530-8

[10] Shan B, Li JY, Liu YJ, et al. LncRNA H19 inhibits the progression of sepsis-induced myocardial injury via regulation of the miR-93-5p/SORBS2 axis. Inflammation. 2021;44(1):344–357. 32996061 10.1007/s10753-020-01340-8

[11] Feng H, Wang JY, Yu B, et al. Peroxisome proliferator-activated receptor-γ coactivator-1α inhibits vascular calcification through sirtuin 3-mediated reduction of mitochondrial oxidative stress. Antioxid Redox Signal. 2019;31(1):75–91. 30829051 10.1089/ars.2018.7620

[12] Chen Y, Lu H, Liu Q, et al. Function of GRIM-19, a mitochondrial respiratory chain complex I protein, in innate immunity. J Biol Chem. 2012;287(32):27227–27235. 22665480 10.1074/jbc.M112.340315 PMC3411064

[13] Wang Y, Luo Q, He X, et al. Emodin induces apoptosis of colon cancer cells via induction of autophagy in a ROS-dependent manner. Oncol Res. 2018;26(6):889–899. 28762328 10.3727/096504017X15009419625178 PMC7844792

[14] Liu TJ, Yeh YC, Lee WL, et al. Insulin ameliorates hypoxia-induced autophagy, endoplasmic reticular stress and apoptosis of myocardial cells: in vitro and ex vivo models. Eur J Pharmacol. 2020;880:173125. 32360347 10.1016/j.ejphar.2020.173125

[15] Onwuli DO, Samuel SF, Sfyri P, et al. The inhibitory subunit of cardiac troponin (cTnI) is modified by arginine methylation in the human heart. Int J Cardiol. 2019;282:76–80. 30772011 10.1016/j.ijcard.2019.01.102

[16] Yan H, Wang H, Zhu X, et al. Adeno-associated virus-mediated delivery of anti-miR-199a tough decoys attenuates cardiac hypertrophy by targeting PGC-1alpha. Mol Ther Nucleic Acids. 2021;23:406–417. 33473326 10.1016/j.omtn.2020.11.007 PMC7787996

[17] Lagan J, Naish JH, Simpson K, et al. Substrate for the myocardial inflammation–heart failure hypothesis identified using novel USPIO methodology. Cardiovascular Imaging. 2021;14(2):365–376. 32305466 10.1016/j.jcmg.2020.02.001 PMC7854561

[18] Zhang Y, Song Z, Huang S, et al. Aloe emodin relieves Ang II-induced endothelial junction dysfunction via promoting ubiquitination mediated NLRP3 inflammasome inactivation. J Leukoc Biol. 2020;108(6):1735–1746. 32573820 10.1002/JLB.3MA0520-582R PMC7754316

[19] Su ZL, Hang PZ, Hu J, et al. Aloe-emodin exerts cholesterol-lowering effects by inhibiting proprotein convertase subtilisin/kexin type 9 in hyperlipidemic rats. Acta Pharmacol Sin. 2020;41(8):1085–1092. 32203084 10.1038/s41401-020-0392-8 PMC7470781

[20] Ren GD, Cui Y, Li WL, et al. Research on cardioprotective effect of irbesartan in rats with myocardial ischemia-reperfusion injury through MAPK-ERK signaling pathway. Eur Rev Med Pharmacol Sci. 2019;23(12):5487–5494. 31298402 10.26355/eurrev_201906_18218

[21] Zhang J, Wang L, Xie W, et al. Melatonin attenuates ER stress and mitochondrial damage in septic cardiomyopathy: a new mechanism involving BAP31 upregulation and MAPK-ERK pathway. J Cell Physiol. 2020;235(3):2847–2856. 31535369 10.1002/jcp.29190

